# Assessment of Cortical Plasticity in Schizophrenia by Transcranial Magnetic Stimulation

**DOI:** 10.1155/2021/5585951

**Published:** 2021-12-02

**Authors:** Turki Abualait, Sultan Alzahrani, Ahmed AlOthman, Fahad Abdulah Alhargan, Nouf Altwaijri, Rooa Khallaf, Eman Nasim, Shahid Bashir

**Affiliations:** ^1^College of Applied Medical Sciences, Imam Abdulrahman Bin Faisal University, Dammam, Saudi Arabia; ^2^Neuroscience Center, King Fahad Specialist Hospital Dammam, Dammam, Saudi Arabia; ^3^Collage of Medicine, King Saud Bin Abdulaziz University for Health Sciences, Riyadh, Saudi Arabia; ^4^College of Medicine, King Saud University, Riyadh, Saudi Arabia

## Abstract

Neural plasticity refers to the capability of the brain to modify its structure and/or function and organization in response to a changing environment. Evidence shows that disruption of *neuronal plasticity* and altered functional connectivity between distinct brain networks contribute significantly to the pathophysiological mechanisms of schizophrenia. Transcranial magnetic stimulation has emerged as a noninvasive brain stimulation tool that can be utilized to investigate cortical excitability with the aim of probing neural plasticity mechanisms. In particular, in pathological disorders, such as schizophrenia, cortical dysfunction, such as an aberrant excitatory-inhibitory balance in cortical networks, altered cortical connectivity, and impairment of critical period timing are very important to be studied using different TMS paradigms. Studying such neurophysiological characteristics and plastic changes would help in elucidating different aspects of the pathophysiological mechanisms underlying schizophrenia. This review attempts to summarize the findings of available TMS studies with diagnostic and characterization aims, but not with therapeutic purposes, in schizophrenia. Findings provide further evidence of aberrant excitatory-inhibitory balance in cortical networks, mediated by neurotransmitter pathways such as the glutamate and GABA systems. Future studies with combining techniques, for instance, TMS with brain imaging or molecular genetic typing, would shed light on the characteristics and predictors of schizophrenia.

## 1. Introduction

Schizophrenia is a complex chronic mental health disorder characterized by a positive and negative array of symptoms [1]. Diagnosis of schizophrenia is incomplete unless one of the positive symptoms is present: hallucinations, delusions, and disorganized speech or behaviors [2]. Despite numerous research in neuroimaging, epidemiology, and genetics, treatment of schizophrenia is still not optimal, and its exact etiologic factors remain elusive. For that reason, researchers are attempting to be more specific in treating only a single psychotic symptom [3, 4]. First- and second-generation antipsychotics are the first line of treatment for schizophrenia [5]. Unfortunately, the schizophrenia prognosis offers poor outcomes despite current treatment; patients often remain symptomatic, and 25%-33% of patients are treatment-resistant [6]. In addition, antipsychotics are associated with significant side effects, leading to additional complications in treating the disease [7].

Application of local stimulation of the neocortex based on a neurophysiological can enhance a greater understanding of the complex architecture of cortical anatomy and its correlation with brain functions. Such methods were applied some time ago to explore a wide range of cerebral functions such as sensory, motor, emotional, and subcortical vascular cognitive impairment [8].

Transcranial magnetic stimulation (TMS) is a “non-invasive method for brain stimulation using the magnetic field to stimulate nerve cells in the brain meninges, subsequently inducing an electric current in the brain tissues that produces neuronal depolarization and generate action potentials” [9]. Introduced in 1985 [10], the TMS has been applied to investigate cortical and spinal excitability, as well as treating neurological and psychiatric disorders [9].

TMS therapeutic effects are applied either through single-pulse TMS, repetitive pulse (rTMS), or theta-burst stimulation (TBS). A single pulse measures the motor threshold in an experimental setting [11]. However, rTMS assists in measuring diseases like major depressive disorder [12]. TMS measures cortical excitability by determining the motor threshold of each individual, which is only conducted through stimulation of the motor system using surface EMG electrodes placed over the motor cortex of the target muscle to obtain a muscle response potential (MEP). This investigation was initiated based on the motor corticospinal pathways [13]. Through the development of TMS techniques “paired-pulse,” it was possible to focus more on specific aspects of cortical excitability, such as inhibitory and facilitatory processes of the components of the motor system that causes various neuropsychiatric disorders [14]. I-wave facilitation and long-interval cortical inhibition (LICI) are some of the specific paired-pulse TMS paradigms. LICI helps to assess an initial suprathreshold stimulus combined with a suprathreshold second stimulus. The initial stimulus produces an inhibition of the evoked motor response to the second (test) stimulus when presented at an appropriate interstimulus interval (50–200 ms). The I-wave facilitation measures the descending I-wave volleys induced by magnetic stimulation [15].

TMS is investigated for the treatment of positive schizophrenic symptoms [16]. Neuronal networks of schizophrenic patients are impaired, especially patients not treated with antipsychotic therapy have impaired extrapyramidal motor disorders that include a general disturbance of fine motor skills, manual dexterity, the bimanual coordination deficits of antisaccade eye movements, and psychomotor speed [17]. In addition, cognitive, motor, neurophysiological, and neuropathological systems are responsible for schizophrenia, causing cortical inhibition.

These disorders are associated with an increased subcortical dopaminergic activity leading to decreased activation of inhibitory cortical afferents, ultimately increasing cortical excitability results. In morphological studies of neuropathological changes, the cortical inhibition is mediated by cortical GABAergic interneurons, closing the final inhibitory dysfunction [18, 19]. While schizophrenia was first treated by TMS over two decades ago, the current evidence still failed to provide a clear picture of the effect [20, 21].

The present study is aimed at reviewing and analyzing the current literature available on the efficacy and assessment of brain plasticity index in schizophrenia through TMS protocols.

## 2. Methods

### 2.1. Search Strategy

We searched for studies published in the following electronic databases including the Cochrane Library, PubMed, EMBASE, and Web of Science, using the search terms as follows: e.g., “Schizophrenia” AND “transcranial magnetic stimulation “or “psychiatric Disorders” AND “TMS” or “schizophrenia” AND “brain plasticity Index” or paired pulse stimulation, from inception to September, 2021. In addition, combinations of Medical Subject Headings (MeSH) and text were used. One hundred and twenty-nine articles were selected. One author performed the initial selection of studies based on title and abstract. Then, each potentially eligible selected study was screened against the inclusion criteria. Finally, for 85 studies, three authors independently reviewed and evaluated the full-text articles to decide and determine whether these articles meet the predefined inclusion criteria. Any related disagreement was resolved by discussion. All selected studies collected from the search were critically examined. The studies for the review were grouped according to the paradigms and parameters examined in schizophrenia patients (Table 1).

### 2.2. Motor Threshold (MT)

The motor threshold (MT) is defined as the lowest intensity of TMS, required to provoke a predefined motor-evoked potential (MEP) with a peak-to-peak amplitude of 50 *μ*V in at least 50% of pursued trials (in 5 of 10 trials) [22]. Minimum stimulation intensity that can elicit a motor response of a given amplitude is either from a muscle at rest, called resting motor threshold (RMT), or during a muscle contraction (almost 5-10% of the target muscle's maximum contraction), named active motor threshold (AMT).

Moreover, medications such as carbamazepine and phenytoin increase the MT activities by changing the sodium channel properties which reflect the transsynaptic excitability of corticospinal response neurons, modulated by altering the presynaptic sodium/calcium channel conductivity [23]. Other substances, such as neuroleptics, antidepressants, or benzodiazepines, acting via the modulation of neurotransmitters offer lesser effects [24]. However, increasing evidence shows that changes in the MT glutamatergic transmission can cause MT activities [25].

Table 1 presents the outcomes of numerous studies reviewed aimed at investigating whether RMT changes in patients with schizophrenia compared to healthy control. From the table, few studies offer no differences in RMT between schizophrenic patients and first-degree relatives compared to healthy control [26]. Following the reported evidence, a significantly lower RMT in schizophrenic patients is noted compared to control subjects, which support the evidence that schizophrenia is linked to the deficits in cortical inhibition (CI) [8, 27, 28]. In addition, naturally occurring and drug-induced psychosis share a pathway that can base on dysfunctional glutamatergic mechanisms [27, 28]. Regarding inhibitory rTMS over the premotor cortex in the intervention with low frequency, some studies revealed a lack of increase in the RMT after stimulation in schizophrenic patients compared to controls. The observed TMS changes could be understood as intracortical motor's primary alterations followed by cortical inhibition defects that might be attributed to antipsychotic medication effect, schizophrenia, or the interaction between the condition and medication [29–31]. The aberrant middle prefrontal-motor cortex connectivity mediates motor inhibitory biomarker in schizophrenia [30]. Therefore, schizophrenia patients display abnormal brain responses to rTMS applied to the premotor cortex that relate to minimized motor cortical inhibition [31–33]. A significant reduction of intracortical inhibition was observed in medicated patients compared with unmedicated patients and controls. This difference was more noticeable in the right hemisphere compared with the left [33]. Additionally, schizophrenia is attributed to a reversed pattern of interhemispheric corticospinal excitability [33]. Another study observed differences between the RMT in patients with schizophrenia treated with risperidone (increased RMT) compared to olanzapine (reduced RMT) [34]. Altogether, these studies provide evidence of impaired excitation/inhibition on cortical oscillations in schizophrenia and suggest that schizophrenia might be characterized by an imbalance of interhemispheric corticospinal excitability [31, 33, 35]. Therefore, there are selective alterations of interhemispheric facilitatory dPM–M1 connectivity in schizophrenia [35].

### 2.3. Motor-Evoked Potentials

Merton and Morton [36] note that MEPs during contraction (contracted-MEPs) are bigger in amplitude and shorter in latency than what MEPs attained during full muscular relaxation (relaxed-MEPs). Thus, muscles that act as prime movers for voluntary movement exert the greatest facilitation [37, 38]. MEPs have been noted to increase when a muscle group is in action due to reduced intracortical inhibition [39]. Other researchers have pinpointed that one of the fundamental findings in patients with schizophrenia is impaired cortical inhibition that mainly results from a dysfunction in the transmission of gamma-aminobutyric acid (GABA) [40, 41]. According to one study, 51 schizophrenic patients with control subjects showed an increase in stimulus intensity for maximum MEP (SI-max) with longer MEP latency and no change in amplitude. SI-max was calculated assuming that MEP size reaches the plateau phase of the curve, and this may affect MEP results [29]. In another study, on hand, preference and TMS asymmetry of cortical motor representation, its effect on MEP was tested, and it was found that the MEP threshold or size did not differ between the preferred and nonpreferred hands. However, the preferred limb had a significantly greater number of scalp stimulation sites that generated MEPs [42]. Du et al. elucidate that used magnetic stimulation markers to demonstrate that high MEPs during schizophrenia are due to abnormal connectivity between the motor cortex and other regions of the brain such as the middle prefrontal gyrus that is affected by schizophrenia [30].

Patients with schizophrenia who are medicine-free demonstrate a higher degree of enhanced I-wave inhibition than those taking medication thus implying that their cortical inhibitory GABAergic activity is impaired [15, 43]. Worth noting is that the main factors that influence MEP in schizophrenic patients are the state of medication and stage of illness. MEPs will be more elevated in patients who are not taking medication and those with advanced stages of schizophrenia [36]. Frank et al. conducted a study that demonstrated that medications such as anticonvulsants and antidepressants can significantly prolong the cortical salient period (CSP) thus controlling MEPs [44]. In another study, interhemispheric facilitation was decreased in schizophrenia, though no change was reported in MEP [35]. Furthermore, a study on the effects of antipsychotic medication responses to TMS of the motor cortex in schizophrenia involved group nine of drug naïve patients compared with a group of nine patients on established neuroleptic medication. There was no difference in TMS threshold strength or latency for inducing compound motor-evoked potentials (cMEPs) [45]. Alternatively, one study compared nine drug-free schizophrenic patients with nine healthy subjects, which revealed a reduction in the latency of MEP [46].

### 2.4. Paired-Pulse Paradigms

Corticocortical excitability can be more directly examined by using paired-pulse TMS in which two consecutive TMS stimuli are applied to the brain at different interstimulus intervals (ISIs). The conditioning stimulus (CS) with approximately 80-95% of the RMT is the first, where the cortex of the actual test stimulus (TS) is set at 110-120% of RMT. Thus, the neuromodulatory effect of the CS on the amplitude of TMS-evoked MEPs depends mostly on two factors: the intensity of CS and TS and the interstimulus interval (ISIs) between both types of stimuli [47].

An ISI of 1-5 ms is “short” and produces inhibitory effects with a lower MEP amplitude. An ISI in the interval of 8-20 ms demonstrates facilitatory effects with the MEP amplitude over 1 mV (amplitude height of the TS without previous CS). When using the bilateral stimulation (dual-pulse technique), a significant reduction of the contralateral MEP amplitude was remarked in the untreated schizophrenic patients, revealing that the antipsychotic treatment may repeal the increased transcallosal inhibition (TCI) in schizophrenia [27]. First-degree relatives of schizophrenics were unrelated to the differences in the duration and latency of the ipsilateral CSP, revealing the transcallosal inhibition and the single-pulse technique [26].

#### 2.4.1. Intracortical Inhibition

Intracortical inhibition can identify changes in variable conditions. Administering GABA-A receptor agonists such as lorazepam primarily caused cortical inhibition [48] and might suppress the I-wave facilitation, which is difficult to be detected [23].

#### 2.4.2. Short Interval Intracortical Inhibition (SICI)

A study explored important aspects of the pathophysiological mechanisms using the paired-pulse method, in which (*n* = 30 schizophrenic patients) served as subjects and half were untreated [49]. However, a decrease is noted in cortical inhibition, only significant in the untreated patients compared to controls and no difference in the facilitation [49, 50]. In the treated patients, an inclination to reduced inhibition is perceived compared to the untreated schizophrenia patients [27, 33]. In another study (*n* = 40 schizophrenic patients), it demonstrates primarily less cortical inhibition in schizophrenic patients [51]. However, a study revealed significantly less cortical inhibition after treating schizophrenic patients with antipsychotic drugs when compared to controls [31]. Moreover, subjects at ultrahigh risk of psychosis exhibited a prolonged CSP with no change in SICI compared to healthy controls, whereas patients with schizophrenia showed a prolonged CSP and a diminished SICI [40].

Compared to the controls before and after administering 1 Hz rTMS on schizophrenic patients, there was less cortical inhibition [31]. However, in prepost comparison for the control group, as anticipated by the inhibitory rTMS, a surge of cortical inhibition was noted [31]. Moreover, the post-rTMS patients had an amplitude of MEPs at rest, which was significantly lower, suggesting a lower modulation of excitability in schizophrenia [32]. In another study, in which the schizophrenics were untreated, no significant differences in cortical excitability were observed with the paired-pulse method as compared with healthy controls [28].

Another study used resting-state functional connectivity to report if SICI was linked to functional connectivity between motor and prefrontal areas and symptoms in schizophrenia (rsFC), which included 24 schizophrenia-spectrum patients and 30 healthy controls. The study indicated that the left middle prefrontal gyrus-motor cortex rsFC was linked to both cortical inhibition and schizophrenia symptoms as stronger the rsFC indicates stronger the motor cortical inhibition [30]. A study examined the changes in SICI to the ultrahigh risk of psychosis (UHR), schizophrenia patients (SZ), and healthy controls. When SZ were compared to healthy controls and SZ patients with UHR individuals, there was a reduced inhibition. In contrast, there was no difference in SICI between UHR subjects and healthy controls [40]. Multiple papers tested the changes in SICI in schizophrenia patients and healthy controls result and have reported a consistent decrease in SICI. A hypothesis might be proposed that changes in SICI develop at all stages of schizophrenia [30, 39, 41, 42, 52]. Compared to literature, only one study found that unmedicated schizophrenia patients had higher SICIs than medicated patients did, for which the researchers were unable to explain [43]. Moreover, a study done on drug-free patients with acute psychosis before and after 3 weeks of treatment did not reveal any changes in SICI. In interpreting this data, it is critical to consider that the absence of the control group might explain the lack of change [44].

#### 2.4.3. Intracortical Facilitation

If the interstimulus interval between the 2 pulses is long, i.e., 9-25 ms, a facilitatory effect is observed, referred to as intracortical facilitation (ICF) [40, 43, 45–56]. ICF can constitute more facilitation and weaker inhibition. In approximately 10 ms, the latency of NMDA receptor-mediated excitatory postsynaptic potentials can occur. Regarding the time course of ICF, some studies [40, 49–51, 57] highlight the importance of glutamatergic transmission of ICF. Typically, NMDA receptor antagonists and GABAA agonists mostly decrease ICF [58]. One study showed acceptable reliability of ICF in schizophrenic patients but poor reliability in healthy subjects [59]. Another study noted that when comparing schizophrenic patients with controls, reducing facilitation is noted based on P60 and N100 ICF. Moreover, the ICF is correlated with the positive and negative total score, representing the pathophysiology of the schizophrenia symptoms, possibly associated with the prefrontal GABA and glutamatergic dysfunctions [60]. Several studies investigated ICF among schizophrenic patients. As one study included 16 ultrahigh risk of psychosis (UHR), 17 schizophrenia patients, and 28 healthy controls investigated, the primary motor cortex did not reveal any changes in the ICF [40]. A similar discovery was made as a one-study test of 24 drug-free schizophrenia patients with acute psychosis before and after starting quetiapine for 3 weeks was unable to detect a significant change in ICF [45]. Another study included 43 unmedicated schizophrenia patients and 38 medicated schizophrenia patients with 32 healthy controls, which failed to show any different ICF changes [43]. This is in line with two studies that compared neuroleptic-naive first-episode schizophrenia patients to healthy controls and found no difference in ICF [28, 41].

This is opposite to two studies that found there is a difference in ICF, as one study involved 13 schizophrenia patients and 13 controls of ICF measured after changing their antipsychotic and were followed up for 8-weeks. ICF measurements were done at baseline and 8 weeks posttreatment. The study reported significantly lower ICF at 8 weeks compared to baseline; this could be attributed to the interstimulus interval which was between 7 and 30 ms, and previous studies have been cross-sectional in design, and this might explain the lack of change in ICF between healthy controls and schizophrenia patients [53]. Another study that measured ICF in the context-based action observation in schizophrenia included 39 schizophrenia patients and 28 healthy controls, which revealed a diminished ICF in schizophrenia patients compared to controls. This could be due to schizophrenia patients having difficulty evaluating and interpreting social cues [39].

#### 2.4.4. Long Interval Cortical Inhibition

Long-interval intracortical inhibition (LICI) refers to suppression of cortical neuronal firing, following a paired-pulse TMS, mediated in part by the primary inhibitory neurotransmitter of the central nervous system; gamma-aminobutyric acid (GABA) receptor B. LICI can be measured either from motor-evoked potentials (MEPs) in the interossei muscles of the hand or directly recorded from the cortex using a concomitant electroencephalography (EEG) method.

Several studies examined the prefrontal cortex, motor cortex TMS-EMG LICI in patients with schizophrenia (SCZ). In a study including 18 SCZ patients (9 were medicated with antipsychotics) and 18 HC, Fitzgerald et al., in a study including 18 patients with schizophrenia (9 were medicated with antipsychotics) and 18 healthy controls, showed that there was no significant difference across both groups related to LICI, suggesting that no influence of neuroleptic medication [15]. Similarly, a cross-sectional study including 54 patients with schizophrenia and 45 healthy controls failed to find a significant LICI difference in patients compared to healthy participants. In addition, there was no significant correlation between LICI and social cognition outcome measures in both groups [61].

It is noteworthy to mention that the studies investigating LICI in the motor cortex of patients with schizophrenia failed to exhibit any significant correlation between LICI changes and neural correlates of schizophrenia symptoms. However, studies examining LICI in the dorsolateral prefrontal cortex (DLPFC) of patients with schizophrenia revealed significant findings. This suggests that altered inhibitory process related to DLPFC, and not to motor cortex, might be underlying the deficits of cognitive function that exist in patients with schizophrenia [61–66].

#### 2.4.5. Cortical Silent Period (CSP)

The application of TMS can assist in studying the transcallosal connection between primary motor cortices (M1) in both human hemispheres, which include transcallosal inhibition (TCI) and facilitation (TCF) with onset latency, typically (30-40 ms). Moreover, the latency and duration of the TCI are known as the transcortical conduction time (transcallosal conduction time, TCT) [50]. The resulting phenomenon is called a cortical silent period (CSP) or a contralateral silent period. By comparing the ipsilateral and contralateral CSP, the contralateral CSP measures the breaking of the target muscle activity of the contralateral side, as per the anatomy of the motor system (pyramidal tract crossing) [48]. The control loops and mechanisms, which produced the CSP principles, are intricate. This involves pallidus, having different cortical and subcortical (globus, the thalamus) structures, facilitated by GABA-B receptors and offered a vital role in neural modulation [67] and via the posterior midbody of the corpus callosum [68].

A previously early study, on the CSP in schizophrenia, revealed no significant differences in the duration of CSP between schizophrenic patients and controls [46]. However, a longer initial latency (onset latency) in treated patients was recorded [45, 56, 69]. Multiple studies reported a consistent shorter CSP in patients without medication effects (risperidone and olanzapine) [34, 39, 52, 70, 71]. On contrary, a different study found a shorter CSP when compared to controls, shown only in the untreated patients. On the other hand, a prolonged CSP is shown in the neuroleptic-treated patients [27, 48]. Another paper found a longer CSP in schizophrenic patients regardless of the application of antipsychotic medication when compared to the control [28]. Another similar study demonstrates a significant CSP prolongation after rTMS in untreated and treated patients compared to controls [31]. One study that explored first-degree relatives of schizophrenia showed no differences in the CSP compared to controls [26].

## 3. Therapeutic Application of TMS

TMS is a noninvasive procedure that uses a magnetic field to stimulate nerve cells of the brain. For the past few decades, TMS has been established as a potential treatment for schizophrenia. TSM enhances casual relationships between the brain and behavior. Furthermore, rTMS is known for its positive effects on neuroplasticity. Given its neuroprotective effects, rTMS can benefit patients with various neuropsychiatric disorders in a short period [1, 14–21, 24–30, 42, 72–76], which include, mood disorders such as depression, schizophrenia, anxiety, dysphemia, autism, substance use disorders, attention-deficit/hyperactivity disorder, and monosymptomatic nocturnal enuresis. The outcomes of a meta-analysis conducted by Freitas et al. showed a significant and moderate effect of rTMS on the negative and positive schizophrenia symptoms. However, the sham-controlled studies revealed that these symptoms demonstrate a small nonsignificant effect [77].

## 4. Discussion

Investigating cortical excitability (motor-neural transmission, ipsilateral recurrence and contralateral CSP, short- or long-latency-intracortical inhibition, and interhemispheric inhibition) can measure cortical inhibition. In patients with schizophrenia, TMS is an auspicious approach to record changes in cortical excitability and to elucidate the underlying pathophysiological mechanisms of the schizophrenia [77–79].

Methodological issues can cause measurement inaccuracies (Table 1). In addition, distinct characteristics and the existing physiological arousal significantly influence the measurement results, such as age [27, 32–34], handedness [75], vigilance [9], state of relaxation [28], gender and time during the menstrual cycle [63], and expectation [13, 14, 68, 72, 76–81]. Lack of compulsory uniform definitions may lead to the formation of automated measuring methods, in which the silent period shows an improvement [78].

Increased MEP is a major observation among patients with schizophrenia due to interference in the cortical inhibitory mechanism [36–39]. MEP is further increased in the absence of medication and when the disease has progressed [36]. Researchers have indicated that dysfunction in the transmission of the inhibitory neurotransmitter GABA and disrupted connections between the motor cortex and other brain regions are the most likely mechanisms [30, 40, 41].

In several lines of evidence, cortical inhibition was observed in schizophrenic patients compared to controls [15, 26–28]. This finding is consistent with the proposed increase in subcortical dopaminergic activity and decrease cortical GABAergic activity. Furthermore, the ICF role in the pathophysiology of schizophrenia needs to be clarified more as a potential indicator to the decrease or increase in the cortical inhibition in a longitudinal study for drug-naive first-episode schizophrenia patients who begin new antipsychotic drug treatment [53].

Confirmed through imaging and TMS technique, schizophrenia patients are suspected to have other neurophysiological and neuropsychological considerations, impaired by interhemispheric transfer [80]. Despite the heterogeneity of the results for the assessment of cortical plasticity with TMS methods, a promising investigation tool is required.

## 5. Conclusion

TMS is an auspicious noninvasive method that can be used to study detailed aspects of cortico-motor-neural excitability in schizophrenia on a pathophysiological basis, with a focus on detecting inhibitory dysfunction (paired-pulse method, transcallosal inhibition, or cortical silent period). Combining it with other methods, like molecular genetic typing, neuropsychological examination, structural and functional imaging including magnetic resonance-volumetric, event-related potentials, and functional magnetic resonance imaging techniques, might contribute to an understanding of future neurobiological vulnerability about schizophrenia population.

## Figures and Tables

**Table 1:** Cortical excitability in schizophrenia studies using transcranial magnetic stimulation.

Study name	Year of publication	Study type	Sample size	Comments
A transcranial magnetic stimulation study of abnormal cortical inhibition in schizophrenia [15]	Fitzgerald, Paul B et al. 2003	Case/control	*N* = 27 18 SCZ	I-wave facilitation 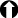 , RMT and LICI = no change
Cortical inhibition in first-degree relatives of schizophrenic patients assessed with transcranial magnetic stimulation [26]	Saka, Meram Can et al. 2005	Case/control	*N* = 26 12 first-degree relatives of SCZ	Three of the 12 healthy relatives lacked transcallosal inhibition (TI)
Evidence for impaired cortical inhibition in schizophrenia using transcranial magnetic stimulation [27]	Daskalakis, Zafiris J et al. 2002	Case/control	*N* = 45 30 SCZ	Cortical inhibition 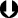
Cortical excitability in neuroleptic-naive first-episode schizophrenic patients [28]	Eichhammer, Peter et al. 2004	Case/control	*N* = 42 21 SCZ	MT 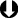 and intracortical inhibition and intracortical facilitation = no change
Cortical motor neurophysiology of patients with schizophrenia: a study using transcranial magnetic stimulation [29]	(Soubasi et al., 2010)	Case/control	*N* = 102 51 SCZ	RMT= 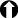 Stimulus intensity for maximum MEP (SI-max)= 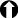
Aberrant middle prefrontal-motor cortex connectivity mediates motor inhibitory biomarker in schizophrenia [30]	(Du et al., 2019)	Case/control	*N* = 54 24 SCZ	SICI = 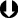 Test stimulation (TS) alone MEP and RMT = no change
Reduced plastic brain responses in schizophrenia: a transcranial magnetic stimulation study [31]	Fitzgerald, Paul B et al. 2004	Case/control	*N* = 44 26 SCZ	Cortical silent period (CSP) and cortical inhibition 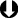
Repetitive transcranial magnetic stimulation reveals abnormal plastic response to premotor cortex stimulation in schizophrenia [32]	Oxley, Tom et al. 2004	Case/control	*N* = 24 12 SCZ	CI 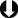 and RMT = no change
Motor cortical excitability in schizophrenia [33]	Pascual-Leone, Alvaro et al. 2002	Case/control	*N* = 21 14 SCZ	RMT 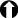 and intracortical inhibition 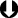
A transcranial magnetic stimulation study of the effects of olanzapine and risperidone on motor cortical excitability in patients with schizophrenia [34]	Fitzgerald, Paul B et al. 2002	Case/control	*N* = 62 40 SCZ	Silent period and transcallosal inhibition 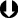
Impaired inter-hemispheric facilitatory connectivity in schizophrenia [35]	(Ribolsi et al., 2011)	Case/control	*N* = 45 16 SCZ medicated, and 9 SCZ unmedicated	RMT and MEP = no change Interhemispheric facilitation 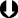 in medicated SCZ
Abnormalities of inhibitory neuronal mechanisms in the motor cortex of patients with schizophrenia [42]	Bajbouj, M et al. 2004	Case/control	*N* = 32 16 SCZ	Postexcitatory inhibition 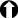 and transcallosal inhibition 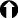
Transcallosal inhibition and motor conduction studies in patients with schizophrenia using transcranial magnetic stimulation [43]	Boroojerdi, B et al.1999	Case/control	*N* = 10 10 SCZ	Transcallosal conduction time (TCT) and duration of the inhibition 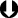
Prolonged cortical silent period among drug-naive subjects at ultra-high risk of psychosis [44]	(Tang et al., 2014)	Case/control	*N* = 61 17 SCZ	SICI= 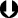 CSP= 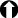 ICF = no change
Antipsychotic treatment with quetiapine increases the cortical silent period [45]	(Frank et al., 2014)	Case	*N* = 24 Drug-free then drugs after 3 weeks	CSP= 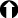 MT, SICI, and intracortical facilitation (ICF) = no change
Effect of antipsychotics on cortical inhibition using transcranial magnetic stimulation [46]	Daskalakis, Zafiris J et al. 2003	Case	*N* = 27	Cortical inhibition = no change
Effects of antipsychotic medication on electromyographic responses to transcranial magnetic stimulation of the motor cortex in schizophrenia [47]	Davey, N J et al. 1997	Case	*N* = 18 *N* = 9 (drug naïve) and *N* = 9 (medicated) SCZ	MEP and MT = no change
Disrupted central inhibition after transcranial magnetic stimulation of motor cortex in schizophrenia with long-term antipsychotic treatment [48]	(Ahlgrén-Rimpiläinen et al., 2013)	Case/control	*N* = 20 11 SCZ	MEP = no change Multiple CSPs were found predominantly in subjects with schizophrenia
A study of transcallosal inhibition in schizophrenia using transcranial magnetic stimulation [49]	Fitzgerald, P B et al. 2002	Case/control	*N* = 45 25 SCZ	Transcallosal inhibition 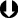
Impairments in motor-cortical inhibitory networks across recent-onset and chronic schizophrenia: a cross-sectional TMS study [50]	(Strube et al., 2014)	Case/control	*N* = 142 41 recent-onset SCZ 42 chronic SCZ	SICI= 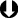 and CSP = 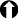
Deficient inhibitory cortical networks in antipsychotic-naive subjects at risk of developing first-episode psychosis and first-episode schizophrenia patients: a cross-sectional study. [51]	(Hasan et al., 2012)	Case/control	*N* = 54 18 SCZ	Intracortical facilitation (ICF) = no change Short-latency intracortical inhibition = 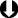 CSP= 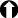
Diminished modulation of motor cortical reactivity during context-based action observation in schizophrenia [40]	(Bagewadi et al., 2019)	Case/control	*N* = 67 39 SCZ	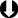 SICI and ICF paradigms. 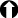 MNS-activity (% cortical reactivity facilitation)
Investigating cortical inhibition in first degree relatives and probands in schizophrenia [52]	(Radhu et al., 2017)	Case/control	*N* = 129 19 SCZ 49 control	Cortical inhibition= 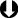
Neural noise and cortical inhibition in schizophrenia [43]	(Carment et al., 2020)	Case/control	*N* = 67 25 SCZ	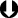 cortical inhibition
Increased short-interval intracortical inhibition in un-medicated patients with schizophrenia [54]	(Schecklmann et al., 2018)	Case/control	*N* = 130 *N* = 43 unmedicated SCZ *N* = 38 Medicated SCZ	SICI= 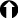 in unmedicated SCHZ RMT, active motor threshold, CSP, and intracortical facilitation = no change
The relationship of the change in symptoms and cognitive functions with the change in cortical inhibition parameters measured by transcranial magnetic stimulation: an eight-week follow-up study [55]	(Yıldız et al., 2015)	Case/control	*N* = 26 13 SCZ	Intracortical facilitation (ICF) is weaker Ipsilateral silent period (ISP) = 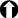 and CI = 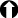
An investigation of motor function in schizophrenia using transcranial magnetic stimulation of the motor cortex [45]	Puri, B K et al. 1996	Case/control	*N* = 18 9 SCZ	Latency of MEP 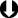 , RMT and MEP = no change
Abnormalities in the evoked frontal oscillatory activity of first-episode psychosis: a TMS/EEG study [60]	(Ferrarelli et al., 2019)	Case/control	*n* = 27 16 SCZ (first episode psychosis)	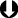 EEG beta/low gamma range oscillations after TMS of M1
Decreased interhemispheric connectivity and increased cortical excitability in unmedicated schizophrenia: a prefrontal interleaved TMS fMRI study [61]	(Webler et al., 2020)	Case/control	*N* = 41 19 SCZ	Hyperexcitability in left BA9 and impaired interhemispheric functional connectivity compared to controls
Electrophysiological responses to transcranial magnetic stimulation in depression and schizophrenia [73]	Abarbanel, J M et al.1996	Case/control	*N* = 20 10 SCZ	MT 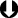 and MEP 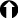

## Data Availability

The data used to support the findings of this study are included in the article.
